# Supplementary material for: Mosquito Cell-Derived Japanese Encephalitis Virus-Like Particles Induce Specific Humoral and Cellular Immune Responses in Mice
Source: Viruses. 2020 Mar 19;12(3):336. doi: 10.3390/v12030336 (PMC7150764; doi:10.3390/v12030336)
Supplement: Supplementary file 1 [file viruses-12-00336-s001.pdf]

Supplementary data

Figure S1. The sequence of the synthetic hr1pag1-JEV prME DNA fragment. This fragment contains the promoter region hr1 and pag1 (shown in light blue and blue, respectively), the coding region (an insect codon-optimized JEV prME gene shown in green from 105-794th amino acid of **AAB66485**) and a poly(A) site (shown in brown). Junction regions shown in black. The underline regions on both ends show restriction sites of *NheI* and *NotI*.

gctagcgtgttttacaagtagaattctacccgtaaagcgagtttagttttgaaaaacaaatgacatcatttgtataatgacat
catcccctgattgtgttttacaagtagaattctatccgtaaagcgagttcagttttgaaaacaaatgagtcatacctaaacac
gttaataatcttctgatatcagcttatgactcaagttatgagccgtgtgcaaaacatgagataagtttatgacatcatccact
gatcgtgcgttacaagtagaattctactcgtaaagccagttcggttatgagccgtgtgcaaaacatgacatcagcttatgac
tcatacttgattgtgttttacgcgtagaattctactcgtaaagcgagttcggttatgagccgtgtgcaaaacatgacatcagc
ttatgagtcataattaatcgtgcgttacaagtagaattctactcgtaatactcatcgaccaatggcgtcgctcggttcttatcg
caacagagtgggggccatccgcactataaaaagccgagactggtgacgaacaccatcagtctgattcgagtcgtgttcat
accgagatctccaccatgggtggcaacgagggttccatcatgtggctggcttccctggctgtggtcatcgcttgcgccggtg
ctatgaagctgtctaacttccagggcaagctgctgatgactatcaacaacaccgacatcgctgacgtgatcgtcatccctac
ttcaaagggagaaaacaggtgctgggtgcgtgctatcgacgtcggttacctgtgcgaggacactatcacctacgaatgcc
caaagctgaccatgggcaacgaccctgaggacgtggactgctggtgcgacaaccaggaagtgtacgtccagtacggaa
ggtgcactaggaccagacactccaagagaactcgccgttcagtgtccgtccagacccacggtgaatccagcctggtcaac
aagaaggaagcttggctggacagcactaaggccacccgctacctgatgaagaccgagaactggatcatccgtaaccctg
gatacgctttcctggctgccgtgctgggatggatgctgggttctaacaacggccagcgcgtggtcttcactatcctgctgct
gctggtcgcccccgcttactcattcaactgcctgggtatgggcaaccgtgacttcatcgagggtgcttccggtgctacctgg
gtggacctggtcctggaaggcgacagctgcctgactatcatggctaacgacaagccaaccctggacgtgcgcatgatcaa
catcgaggcttctcagctggccgaagtccgttcatactgctaccacgcttctgtgactgacatctcaaccgtcgccaggtgc
cctaccactggagaggctcacaacgaaaagagagccgactcttcatacgtgtgcaagcagggtttcaccgacaggggat
ggggtaacggctgcggactgttcggcaagggcagcatcgacacttgcgctaagttctcttgcacctcaaaggccatcggta
gaactatccagcccgagaacatcaagtacgaagtgggtatcttcgtccacggcaccactacctccgagaaccacggaaac
tactctgctcaagtgggtgcctcacaggctgccaagttcactgtcaccccaaacgctccttccatcactctgaagctgggag
actacggagaggtgaccctggactgcgaaccaaggagcggcctgaacaccgaggccttctacgtgatgactgtcggaag
caagtctttcctggtccacagagaatggttccacgacctggctctgccctggacttctccttcttctactgcttggaggaaca
gggagctgctgatggaattcgaggaagcccacgctaccaagcagtccgtggtcgctctgggttctcaagagggaggtctc
caccaggccctggctggagctatcgtggtcgaatactcttcatccgtgaagctgacttctggccacctgaagtgcaggctg
aagatggacaagctggccctgaagggaactacctacggaatgtgcaccgagaagttctcattcgctaagaaccccgccg
acactggacacggtaccgtggtcatcgaactgtcatactccggcagcgacggaccttgcaagatccccatcgtgtccgtcg
cttccctgaacgacatgacccccgtgggacgcctggtgactgtcaacccattcgtcgctaccagctcagccaactctaagg
tgctggtcgagatggaacctcccttcggtgactcatacatcgtgatcggccgcggagacaagcagatcaaccaccactgg
cacaaggctggctccactctgggaaaggccttcagcactaccctgaagggtgctcaacgtctggctgctctgggcgacac

tgcttgggacttcggatctatcggcggagtgttcaactcaatcggcaaggctgtgcaccaggtcttcggtggcgccttccgt
actctgttcggaggtatgtcctggatcacccagggtctgatgggcgctctgctgctgtggatgggtgtgaacgccagggac
agaagcatcgctctggccttcctggctactggcggagtgctggtcttcctggctaccaacgtccacgcctaactgcagagta
gatgccgaccgaacaagagctgatttcgagaacgcctcagccagcaactcgcgcgagcctagcaagttgtttattgcagct
tataatggttacaaataaagcaatagcatcacaaatttcacaaataaagcatttttttcactgcattctagttgtggtttgtcc
aaactcatcaatgtatcttatcatgtctggatcggggcggccgc
